# Recognition and penetration of the HIV-1 Env glycan shield by potent broadly neutralizing antibodies

**DOI:** 10.1186/1742-4690-9-S2-P48

**Published:** 2012-09-13

**Authors:** J Julien, L Kong, R Pejchal, R Khayat, J Lee, RS Stanfield, LM Walker, KJ Doores, E Folkowska, P Poignard, R Depetris, RW Sanders, WC Koff, JP Moore, AB Ward, DR Burton, IA Wilson

**Affiliations:** 1 Dept. of Molecular Biology, The Scripps Research Institute, La Jolla, CA, USA; 2 Dept Imm & Microbial Sci & IAVI NAC, The Scripps Research Institute, La Jolla, CA, USA; 3 The Scripps Research Institute, Ragon Institute MIT, Boston, MA, USA; 4 Weill Medical College of Cornell University, New York, NY, USA; 5 Weill Medical College of Cornell University, Academic Medical Center, Amsterdam, the Netherlands; 6 The International AIDS Vaccine Initiative, New York, NY, USA; 7 Dept. Imm & Microbial Sci and IAVI NAC TSRI, Ragon Institute MIT, USA; 8 The Scripps Research Institute, La Jolla, CA, USA

## Background

Human monoclonal antibodies have been characterized recently that potently neutralize HIV-1 isolates across all clades. These exciting new antibodies (PGT series) were derived from direct functional screening of B cells from IAVI protocol G donors (Theraclone/Monogram) and are unusually potent with binding predicted to be to novel glycan-dependent epitopes on Env.

## Methods

Structures of these new PGT antibodies are being determined by x-ray crystallography and electron microscopy with further characterization using binding and mutagenesis assays.

## Results

The crystal and EM structures so far have been elucidated for many of these antibodies. Work on the others are in progress, focusing on Fab complexes with glycans, gp120 core and fragment constructs, as well as Env trimers.

## Conclusion

Structural characterization and biochemical analysis of these antibodies have uncovered novel specificities to new glycan-dependent epitopes and reveal further mechanisms for viral neutralization. These new epitopes provide additional insights for neutralization of HIV-1 and how antibodies can bind and penetrate the glycan shield, a novel framework for structure-assisted vaccine design.

This study was supported by the International AIDS Vaccine Initiative (IAVI), Ragon Institute of MGH, MIT and Harvard, and NIH AI84817.

